# A Comparative Analysis of Multipotent Mesenchymal Stromal Cells derived from Different Sources, with a Focus on Neuroregenerative Potential

**DOI:** 10.1038/s41598-020-61167-z

**Published:** 2020-03-09

**Authors:** Yuriy Petrenko, Irena Vackova, Kristyna Kekulova, Milada Chudickova, Zuzana Koci, Karolina Turnovcova, Helena Kupcova Skalnikova, Petr Vodicka, Sarka Kubinova

**Affiliations:** 1 Institute of Experimental Medicine of the Czech Academy of Sciences, Videnska 1083, 14220 Prague, Czech Republic; 2 2nd Medical Faculty, Charles University, V Uvalu 84, 15006 Prague, Czech Republic; 3 Institute of Animal Physiology and Genetics of the Czech Academy of Sciences, Rumburska 89, 277 21 Libechov, Czech Republic

**Keywords:** Neurotrophic factors, Mesenchymal stem cells

## Abstract

Multipotent mesenchymal stromal cells (MSCs) can be considered an accessible therapeutic tool for regenerative medicine. Here, we compared the growth kinetics, immunophenotypic and immunomodulatory properties, gene expression and secretome profile of MSCs derived from human adult bone marrow (BM-MSCs), adipose tissue (AT-MSCs) and Wharton’s jelly (WJ-MSCs) cultured in clinically-relevant conditions, with the focus on the neuroregenerative potential. All the cell types were positive for CD10/CD29/CD44/CD73/CD90/CD105/HLA-ABC and negative for CD14/CD45/CD235a/CD271/HLA-DR/VEGFR2 markers, but they differed in the expression of CD34/CD133/CD146/SSEA-4/MSCA-1/CD271/HLA-DR markers. BM-MSCs displayed the highest immunomodulatory activity compared to AT- and WJ-MSCs. On the other hand, BM-MSCs secreted the lower content and had the lower gene expression of neurotrophic growth factors compared to other cell lines, which may be caused by the higher sensitivity of BM-MSCs to nutrient limitations. Despite the differences in growth factor secretion, the MSC secretome derived from all cell sources had a pronounced neurotrophic potential to stimulate the neurite outgrowth of DRG-neurons and reduce the cell death of neural stem/progenitor cells after H_2_O_2_ treatment. Overall, our study provides important information for the transfer of basic MSC research towards clinical-grade manufacturing and therapeutic applications.

## Introduction

Multipotent mesenchymal stromal cells (MSCs) can be considered a unique and accessible therapeutic tool for regenerative medicine and tissue engineering. Progress in using MSCs for the treatment of various disorders is continuously growing and the scope for their potential application is becoming wider. The broad spectrum of pathologies where MSCs can be applied, either as an injectable suspension or within a scaffold or hydrogels, has been reported elsewhere and includes wounds^1,2^, bone and cartilage defects^3^, graft-versus-host disease^4^, cardiovascular^5,6^ or neural disorders (multiple sclerosis, stroke, spinal cord injury etc.)^7–9^. Previously we have shown that MSC transplantation has a positive impact on neural tissue regeneration in spinal cord injury models^8,10,11^ or a protective effect in amyotrophic lateral sclerosis (SOD1G93A) rats^7^. There is also growing evidence confirming the high therapeutic potential of MSC-derived secretome as an alternative cell-free treatment of spinal cord injury^12^, Parkinson’s disease^13^, or Alzheimer’s disease models^14^. In this case, the source of the MSCs for cell transplantation or the preparation of secretome-based therapeutics may determine the profile of molecules involved in the neurotrophic/neuroprotective activity of cells.

Cells with MSC-like properties have been already isolated from the variety of adult and perinatal human tissues, including but not limited to bone marrow, adipose tissue, skin, dental pulp, peripheral or umbilical cord blood, placenta, umbilical cord Wharton’s jelly (recently reviewed in^15^). The correspondence of MSCs from different sources to the minimal criteria proposed by the International Society for Cellular Therapy (ISCT) does not confirm their equivalence in functional properties and paracrine activity. Developmental stage, cell niche organization and the functional predisposition of the source tissue can be considered as innate factors, which may determine the paracrine and differentiation properties of MSCs. Additional “acquired” factors occur after cell isolation and following processing. The composition of culture medium and supplements, substrate properties and oxygen concentration would most likely affect the features of MSCs through different mechanisms^16,17^. Therefore, the ideal way to compare the sources of MSCs in view of further clinical application should be based on the assessment of the specific properties of candidate cell types expanded in similar conditions, starting from the initial stages of cell processing. Previously Fekete *et al*. reported a comprehensive investigation of the effects of culture conditions on the functional properties of MSCs, assigning MEM-alpha medium (α-MEM) supplemented with 5% of human platelet lysate (PL) as the best complete culture medium, which assures optimal proliferation of cells^18^. The application of PL as a culture medium supplement is currently one of the methods of choice for the efficient clinical-grade xeno-free expansion of MSCs, since the “classical” fetal bovine/calf serum (FBS) has been banned by regulatory authorities due to its xenogeneic origin^19–21^. The cocktail of growth factors present in PL preparations has been reported to be a highly efficient mitogen for MSCs, providing enhanced growth capacity while preserving their differentiation properties^19–21^. However, not much information is currently reported on the properties of MSCs derived from different sources after *ex vivo* expansion in a PL-containing medium.

In this study, we show a thorough comparative analysis of the MSC therapeutic potential *in vitro* depending on the tissue of origin. We compared MSCs derived from human adult bone marrow (BM-MSCs), adipose tissue (AT-MSCs) and Wharton’s jelly (WJ-MSCs), focusing on the growth kinetics, immunophenotypic and differentiation properties, immunomodulatory activity, gene expression and secretome content. We further focused on the neurotrophic properties of these cells, and tried to reveal the differences between the studied cell types.

## Results

### Characterization of MSCs derived from different sources

**Figure 1:**
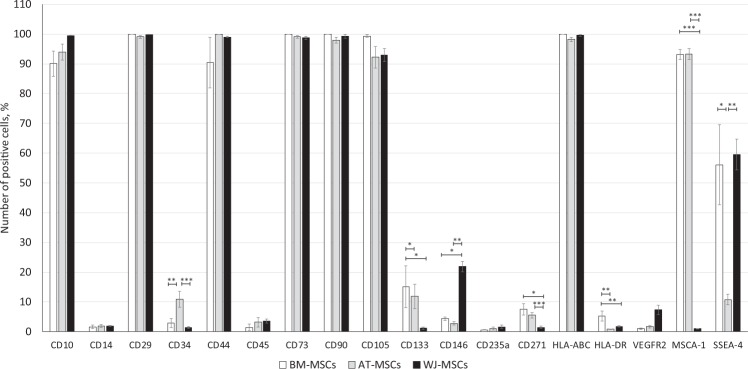
Immunophenotype of MSCs derived from different sources. Data are expressed as mean ± SEM, N = 7 (*p < 0.05; **p < 0.01; ***p < 0.001). One-way ANOVA with Student–Newman–Keuls post hoc pair-to-pair test.

To confirm the identity of cells, we characterized *in vitro* expanded MSCs derived from bone marrow, adipose tissue or Wharton’s jelly by the minimal criteria proposed by ISCT^22^. After isolation, cells attached to the culture plate and attained fibroblast-like morphology specific for MSCs. We confirmed that MSCs from all sources were able to differentiate into adipogenic, osteogenic and chondrogenic lineages (Supplementary information, Fig. S1). The purity of MSC populations was assessed by immunophenotypic profile of cell cultures at passage 3 using flow cytometry (Fig. 1). The positive expression of CD10, CD29, CD44, CD73, CD90, CD105, HLA-ABC was observed in more than 90% of cells, independently of the tissue of origin. The CD271 and HLA-DR were expressed in less than 5–7% of MSC population depending on the tissue of origin. There were no significant differences in the expression of CD14, CD45, CD235a and VEGFR2 markers between MSCs from different sources. All the evaluated cell lines were negative for these markers. Some differences were revealed in the expression of CD133 in BM- and AT-MSCs compared to WJ-MSCs (Fig. 1). A higher expression of CD34 was observed in AT-MSCs (10.9 ± 2.7%), whereas CD146 was notably expressed in WJ-MSCs cultures (21.8 ± 1.7%) and not in AT- and BM-MSCs. There was a huge difference in the expression of MSCA-1 by BM- and AT-MSCs (more than 90% positive cells), compared to WJ-MSCs (no expression). Alternatively, the SSEA-4 was expressed in more than 50% of BM- and WJ-MSCs, compared to 10.7 ± 1.7% in AT-MSCs cultures. Although the majority of cell lines had an immunophenotypic profile corresponding to MSCs (as proposed by ISCT), there were remarkable differences in the expression of several markers, confirming the non-equality of the MSCs, most likely associated with the initial source tissue peculiarities.

### Growth kinetics

**Figure 2:**
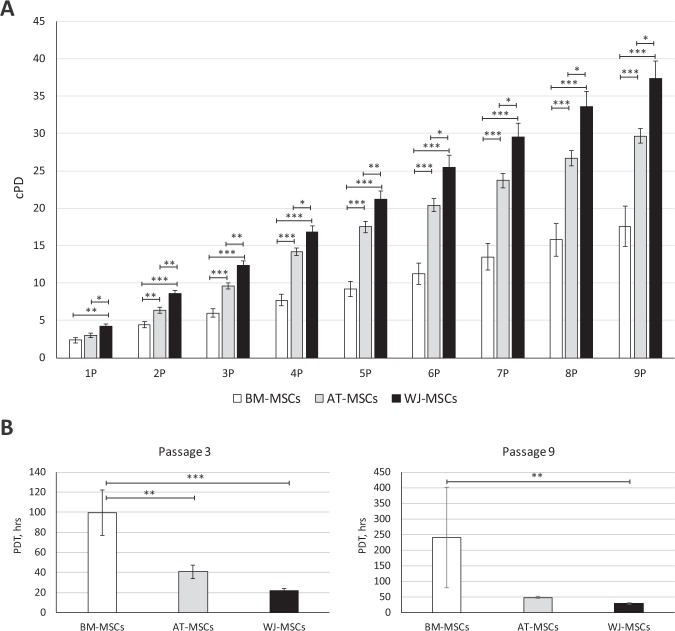
Growth kinetics of MSCs, derived from different sources, assessed by cPD (**A**) and PDT (**B**). Data are expressed as mean ± SEM (N = 7 for BM-MSCs, N = 9 for AT-MSCs and N = 15 for WJ-MSCs). (*p < 0.05; **p < 0.01; ***p < 0.001). A - One-way ANOVA with Student–Newman–Keuls post hoc pair-to-pair test; B - One-way ANOVA with Dunn’s post hoc pair-to-pair test.

The average duration of primary culture before adherent cells reached confluence comprised around 7 and 8 days for AT and BM and 13 days for WJ cells (Table 1). After the first passage, the growth kinetics of cells differed among the sources of cell isolation (Fig. 2). The proliferation rate of BM-derived MSC cultures was significantly lower compared to the other evaluated cell lines. At passage 3, the cumulative population doublings (cPD) of BM-MSCs were 6 ± 0.5, while in AT-MSCs and WJ-MSCs this parameter was significantly higher and reached 9.6 ± 0.4 and 12.3 ± 0.7, respectively (Fig. 2A). Subsequently, the population doubling time (Fig. 2B) was lowest in WJ-MSCs (21 ± 2 hrs) and AT-MSCs (40 ± 7 hrs), remarkably exceeding BM-MSCs (99 ± 22 hrs). Such a tendency in cell growth parameters remained until passage 9 (Fig. 2B). The growth kinetics of MSCs are summarized in Table 1, which shows that the duration needed to achieve a clinically-relevant cell number is 2-fold longer for BM-MSCs, compared to AT-MSCs and WJ-MSCs.

**Table 1:** Summarizing table.

Tissue source	Isolation method (average number of isolated cells)	Average duration (days)*			Passage to reach 140 × 10^6^ cells*	Average PDT (at indicated passages), hrs	Three-lineages differentiation	Immunomodulatory activity	
		Primary culture	Expansion	Total				Contact co-culture (MSC:PBMC ratio)	Transwell co-culture (MSC:PBMC ratio)
**A – General properties of MSCs**									
Bone marrow	Gelofusine sedimentation	8.4	34.6	43.1	4	68–185 hrs	+++	1:10	1:2
Adipose tissue	Collagenase digestion	6.9	13	19.9	2	40–45 hrs	+++	1:5	1:1
Wharton’s jelly	Liberase digestion	13	7.6	20.6	2	19–25 hrs	+++	1:5	>1:1
**B – Neurotrophic/neuroprotective properties of MSCs**									
**Tissue source**	**Secretome profile (growth factors secretion)**					**Neuroptrophic/neuroprotection**			
	**HGF**	**FGF-2**	**VEGF-A**	**NGF**	**BDNF**	**Stimulation neurite outgrowth**	**Protection against oxidative stress**		
Bone marrow	+	+	+++	+	++	++	+++		
Adipose tissue	++	++	++	−	++	+++	+++		
Wharton’s Jelly	+++	++	−	++	+++	+++	+++		

*Theoretical prediction, showing the average time needed to achieve clinically-relevant cell number (2 × 10^6^ cells per kg × 70 kg = 140 × 10^6^ cells), based on the results obtained in the current study. The theoretical prediction of the yield of MSCs that could be obtained utilizing all harvested cells for sub-cultivation was calculated using the formula: Theoretical MSCs yield = Nh1 * 2^cPD^, where Nh1 means cell number after the first cell harvest (passage 1). Data from minimally five independent experiments are reported.

We also confirmed the migration capacity of all MSCs towards SDF-1α in a transwell system. The migration rate did not differ between individual cell types (Supplementary information, Fig. S2).

### Immunomodulatory activity

**Figure 3:**
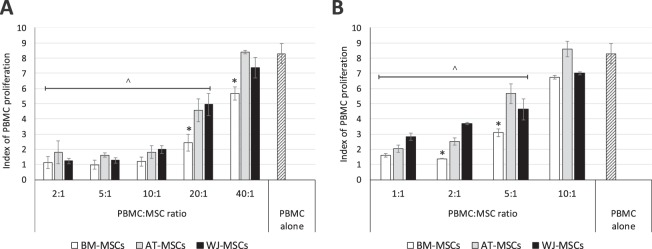
Immunomodulatory properties of MSCs derived from different sources in contact (**A**) or transwell (**B**) co-culture with PBMC (mean ± SEM, N = 3 in duplicates). *p < 0.05 compared to other sources; ^p < 0.05 compared to PBMC alone group. Mann-Whitney nonparametric test.

The immunomodulatory activity of MSCs derived from different sources was assessed by their ability to suppress the phytohaemagglutinin (PHA)-induced proliferation of peripheral blood mononuclear cells (PBMC) in contact (Fig. 3A) or non-contact (transwell) co-culture (Fig. 3B). The activation of PBMC by PHA resulted in an 8.3 ± 0.7 times increase in cell proliferation. In contact co-culture conditions, MSCs from all evaluated sources almost completely inhibited PBMC proliferation at PBMC:MSC ratio 10:1 or lower (Fig. 3A). At higher PBMC:MSC ratios (20:1 and 40:1) the inhibitory potential of BM-MSCs was significantly higher compared to other sources. At ratio 40:1, no visible inhibitory activity was detected in AT- and WJ-MSCs co-cultures with PBMC.

Within the transwell system, which eliminates direct cell-cell contact, MSCs were also able to suppress PHA-induced PBMC proliferation, however at lower PBMC:MSC ratios (Fig. 3B). The most prominent inhibitory activity was observed at 1:1 ratio for all studied groups. At 2:1 ratio the complete inhibition of PBMC proliferation was only observed in BM-MSC cultures. A higher suppressive capacity of BM-MSCs was also detected in a 5:1 co-culture system. At PBMC:MSC ratio 10:1, no significant inhibition of PBMC proliferation was observed in any of the studied cell lines.

These results confirm the capacity of MSCs derived from different sources to suppress immune response through cell-cell interactions and non-contact paracrine mechanisms, with BM-MSCs having the most prominent immunosuppressive potential.

### Secretome composition of MSCs derived from different sources

**Figure 4:**
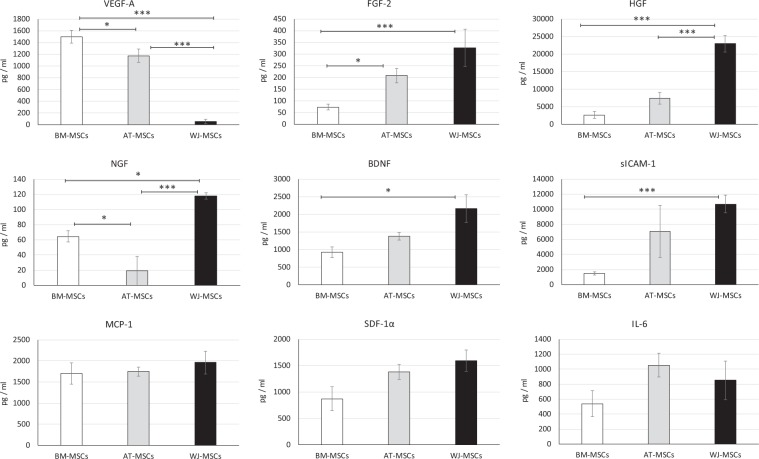
The secretome profile of MSCs of different tissue origin in serum starvation (PL-free) conditions (mean ± SEM, N = 3 in duplicates). *p < 0.05, ***p < 0.001. One-way ANOVA with Student–Newman–Keuls post hoc pair-to-pair test.

To assess the paracrine activity of MSCs in more detail, we analysed the protein composition of the secretomes produced by BM-, AT- and WJ-MSC cultures under PL-free conditions with the focus on angiogenic and neurotrophic factor secretion and inflammatory/anti-inflammatory factors, as well as adhesion molecules (Fig. 4).

There was a noticeable difference in the production of pro-angiogenic/neurotrophic factors (HGF, FGF-2, VEGF-A, BDNF, NGF) by MSCs, depending on their source (Fig. 4). The secretion of HGF was 3 times higher in WJ-MSCs compared to AT-MSCs, and around 9 times higher than in BM-MSCs. The quantity of FGF-2 and NGF in conditioned medium (CM) produced by WJ-MSCs was significantly higher compared to other evaluated cell lines. The most prominent secretion of BDNF also corresponded to WJ-MSC cultures. In contrast, the content of VEGF-A was highest in BM-MSC-derived CM, and lowest in WJ-MSC-CM. MSCs from all evaluated sources produced varying levels of cytokines, adhesion molecules and chemoattractants (IL-6, sICAM-1, SDF-1α and MCP-1). The sICAM-1 production was significantly higher in WJ-MSCs cultures compared to BM-MSCs. No significant differences were detected between the quantities of MCP-1, IL-6 and SDF-1α. The additional hypoxic treatment of MSCs cultured in PL-free conditions resulted in a significant increase of VEGF-A secretion by BM-MSCs and in the production of NGF by AT-MSCs (Table 2).

**Table 2:** The effect of hypoxia on growth factor secretion by serum starved MSCs derived from different sources (pg/ml). Values are expressed as mean ± SEM (duplicates of 3 donors were used for each MSC source).

	BM-MSCs		AT-MSCs		WJ-MSCs	
	21% O_2_	5% O_2_	21% O_2_	5% O_2_	21% O_2_	5% O_2_
VEGF-A	1498 ± 108	2058 ± 112*	1175 ± 114	1348 ± 96	57 ± 36	29 ± 7
FGF-2	74 ± 12	174 ± 45	208 ± 30	252 ± 53	328 ± 79	454 ± 88
HGF	2621 ± 975	3211 ± 1043	7447 ± 1719	5843 ± 767	22950 ± 2368	22684 ± 2449
NGF	65 ± 7	57 ± 19	19 ± 18	80 ± 12*	118 ± 4	113 ± 11
BDNF	927 ± 156	1239 ± 205	1378 ± 99	1274 ± 102	2166 ± 389	2810 ± 314
IL-6	539 ± 171	764 ± 293	1055 ± 158	1035 ± 88	852 ± 259	846 ± 367
sICAM-1	1489 ± 236	1348 ± 454	7078 ± 3451	8517 ± 4264	10693 ± 1154	15137 ± 1981
SDF-1α	870 ± 226	1318 ± 203	1384 ± 141	1270 ± 54	1593 ± 204	1937 ± 225
MCP-1	1699 ± 251	1738 ± 299	1748 ± 111	1608 ± 216	1961 ± 276	1817 ± 109

*Values are significantly different compared to the same group in 21% O_2_ (p < 0.05), One-way ANOVA with with Student–Newman–Keuls post hoc pair-to-pair test.

To reveal the paracrine neurotrophic and neuroprotective activity of MSCs, we assessed the effect of cell-derived secretome on the recovery of neural stem cells after H_2_O_2_ treatment and neurite outgrowth of dorsal root ganglion (DRG)-neurons in functional *in vitro* assays.

### The neuroprotective effect of MSC-CM on neural stem cells after oxidative stress

**Figure 5:**
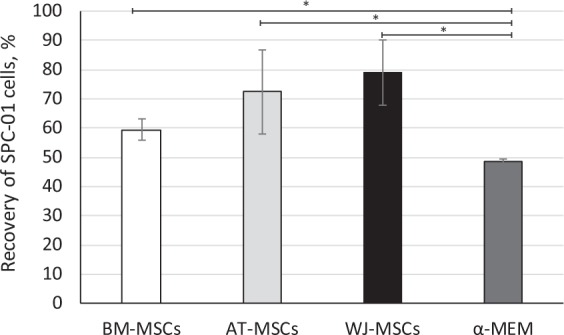
The effect of MSC-derived CM on the recovery of neural stem cells (SPC-01) after H_2_O_2_ treatment (mean ± SEM, N = 3 in duplicates). *p < 0.05 - values are significantly different compared to control. Mann-Whitney nonparametric test.

A significant 10–30% increase in the recovery of SPC-01 neural stem cells after H_2_O_2_ treatment was observed in the presence of a CM produced by MSCs from different sources (Fig. 5). Although there were no significant differences among the tissues of MSC origin, the data confirms the anti-apoptotic properties of MSC-derived secretome.

### The effect of MSC conditioned medium on the neurite outgrowth of DRG-neurons

**Figure 6:**
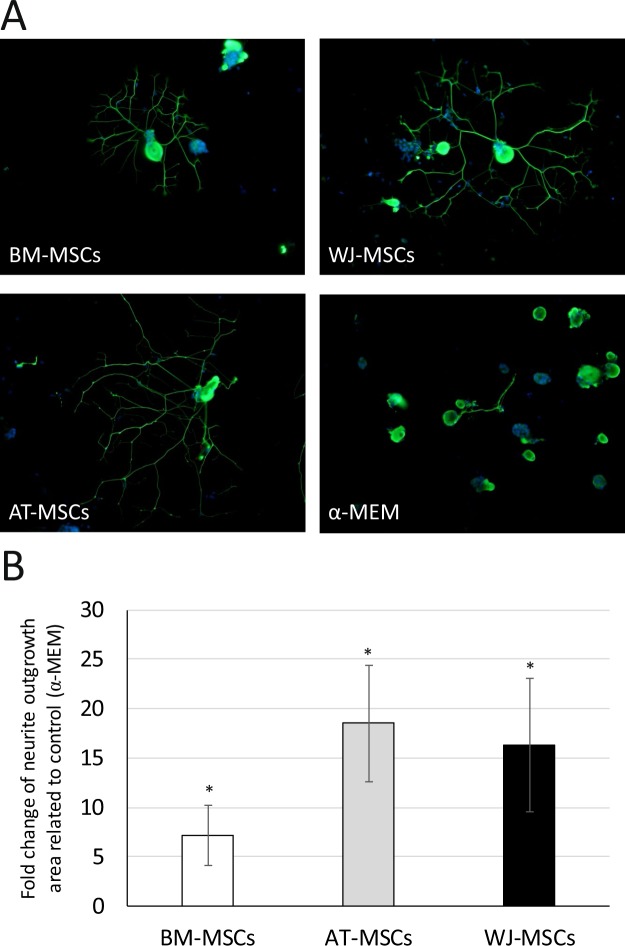
The effect of MSC-derived CM on neurite growth of rat DRG neurons: (**A**) DRG neurons cultured in the presence of conditioned or control medium (beta3 tubulin/DAPI staining, magn. ×200); (**B**) the fold change in mean area (mean ± SEM, N = 3 in duplicates) of neurite growth related to control (α-MEM). *p < 0.05 values are significantly higher compared to control (α-MEM). Mann-Whitney nonparametric test.

The application of secretome of MSCs on dorsal root ganglion (DRG)-neuron cultures resulted in a significant increase of the total area of neurites (Fig. 6) compared to the control (non-conditioned) medium. To reveal the role of individual secreted factors in DRGs functionality, we prepared a screening experiment supplementing the culture medium with HGF, FGF-2 or BDNF at a concentration of 10 ng/ml (Supplementary information, Fig. S3). Independently of the growth factor used, the area of neurite growth was significantly higher than in control conditions.

Although no significant differences were found between the MSC sources due to donor-associated variations, a tendency of lower stimulatory activity towards DRG-neurons was indicated for BM-MSC-derived CM. In both functional *in vitro* assays as well as in proteomic analysis we applied serum starvation (PL-free) culture conditions to selectively distinguish the growth factors and cytokines in MSC-derived secretome from those contained in PL. However, it is known that serum starvation may introduce significant stress into the normal physiology of cells. We hypothesized that the comparatively lower activity of BM-MSC-derived secretome may be connected with the stress induced in cells by serum starvation. To confirm this hypothesis, we studied the overall metabolic activity of MSCs derived from different sources and their gene expression before and after PL-free culture.

### The metabolic activity of MSCs under serum starvation conditions

**Figure 7:**
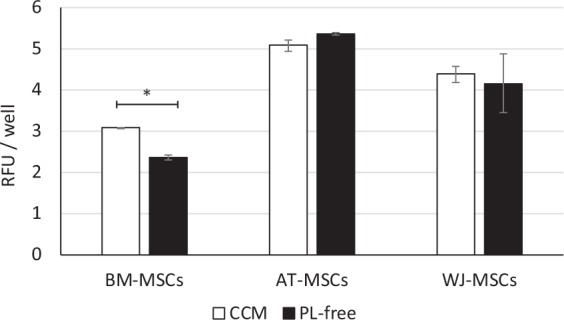
The effect of serum starvation (PL-free) conditions on metabolic activity of MSCs derived from different tissues (mean ± SEM, N = 3 in duplicates). *p < 0.05 values are significantly different compared to CCM group. Paired Student’s t-test.

To assess whether serum starvation affects overall metabolic activity, MSCs from different sources were expanded during 5 days in complete culture medium (CCM) and treated with mitomycin C to stop cell proliferation. The medium was changed again, either to CCM or a serum-free medium without any supplements, to induce serum starvation conditions. After 24 hrs, the metabolic activity of cells was determined by alamar blue (AB). No significant reduction of metabolic activity was observed for AT-MSCs and WJ-MSCs after serum starvation, indirectly confirming the complete preservation of cell viability in stressed conditions. However, a significant decrease in overall metabolic activity was detected in BM-MSCs cultures (Fig. 7).

### Gene expression

**Figure 8:**
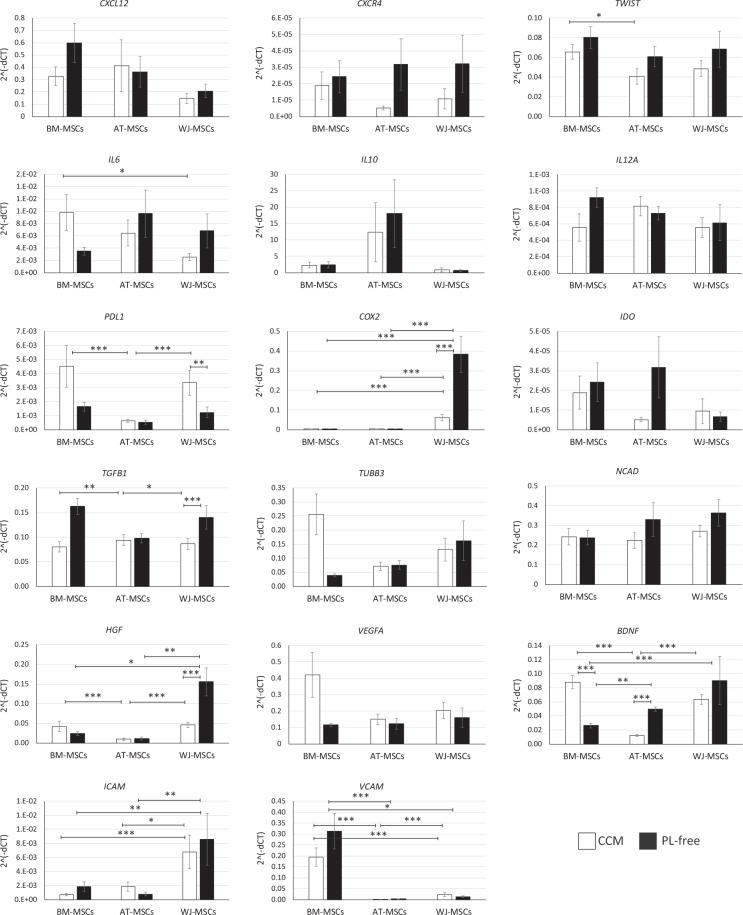
Relative gene expression of *ex vivo* expanded MSCs derived from different tissues cultured in CCM or serum starvation (PL-free) conditions (mean ± SEM, N = 3 in duplicates for PL-free groups, N = 7 in duplicates for CCM groups). *p < 0.05; **p < 0.01; ***p < 0.001). One-way ANOVA.

We analysed the expression of 17 genes in MSCs related to the function: chemokines (*CXCL12, CXCR4*), adhesion molecules (*ICAM, VCAM, NCAD*), transcription factors (*TWIST*); immunoregulatory/inflammatory/anti-inflammatory (*PDL1, COX2, IDO, TGFB1, IL6, IL10, IL12A*), angiogenic/neurotrophic factors (*VEGFA, HGF, BDNF)*, and neural specific marker *TUBB3* (Fig. 8). When cultured in CCM, significant differences between the MSC gene expression depending on tissue sources were indicated for *TWIST, IL6, PDL1, COX2, TGFB1, HGF, BDNF, ICAM* and *VCAM*. The expression of transcription factor *TWIST* was significantly higher in BM-MSCs compared to AT-MSCs. The higher expression of *IL6* was detected in BM-MSCs compared to WJ-MSCs. The expression of *PDL1* was significantly higher in BM- and WJ-MSCs compared to AT-derived cells. A significantly higher expression of *COX2* and *ICAM* was observed in WJ-MSCs compared to other cells. The expression of *TGFB1* was higher in AT-MSCs compared to cells from the other sources. The lowest expression of *HGF* and *BDNF* was observed in AT-MSCs compared to other sources. The expression of *VCAM* was higher in BM-MSCs compared to other cell sources. No significant differences between the three cell sources were then found for *CXCL12, CXCR4, IL10; IL12A, IDO, TUBB3, NCAD* and *VEGFA* expression (Fig. 8). The *VEGFA* and *TUBB3* expression tended to be higher in BM-MSCs, however no statistical significance was found, compared to other cells.

In response to stress (PL-free) conditions, we found significant up- or downregulation of several analysed genes (Fig. 8). Significant upregulation of *COX2*, *TGFB1* and *HGF* and downregulation of *PDL1* was detected for WJ-MSCs. The *BDNF* was upregulated in AT-MSCs. In contrast, all genes associated with neurotrophic activity were either unchanged (HGF) or significantly downregulated (BDNF) in BM-MSCs after PL-free culture (Fig. 8).

When comparing the gene expression between MSCs derived from BM, AT and WJ in PL-free culture conditions, significant differences were found for *HGF*, *BDNF, ICAM, VCAM* and *COX2*. Under serum starvation, the *HGF and COX2* expression was significantly higher in WJ-MSCs compared to other cell sources (Fig. 8). As expected, the level of *BDNF* expression in BM-MSCs was significantly lower compared to other cell lines (Fig. 8). The expression of *ICAM* and *VCAM* genes remained similar after serum starvation (*ICAM* expression was higher in WJ-MSCs, while *VCAM* in BM-MSCs compared to other tissues). No significant differences among the cell sources, after serum starvation, were detected for *CXCL12, CXCR4, TWIST, IL6, IL10, IL12A, PDL1, IDO, TGFB1, TUBB3, NCAD* and *VEGFA*.

## Discussion

In the current study we have thoroughly evaluated the differences and similarities of MSCs derived from human bone marrow, adipose tissue and Wharton’s jelly *in vitro*, using equal cell culture conditions. The results confirm the non-equivalency of the MSCs in growth kinetics *in vitro*, immunophenotype and immunomodulatory properties, as well as their gene expression and secretome content. MSCs derived from all sources had pronounced neurotrophic potential, assessed by secretome composition and paracrine activity *in vitro*. The maintenance of metabolic properties and growth factor production in stress conditions (under serum starvation and/or hypoxia) may indirectly indicate the therapeutic potential of cells after cell transplantation.

To verify the identity of MSCs derived from BM, AT and WJ and to try to compare the generated cell lines, we studied their immunophenotype. According to the marker panel proposed by ISCT, all the cell lines evaluated in our study corresponded to MSCs^22^. However, we observed remarkable differences in the expression of CD34, CD133, CD146, MSCA-1 and SSEA-4. The increased expression of CD34 (hematopoietic stem cells/diverse progenitors/endothelial marker) in AT-MSCs shown in our study confirms the data previously reported elsewhere^23–26^. The common explanation for the CD34 expression in cells from stromal vascular fraction of adipose tissue is the perivascular origin of AT-MSCs^27^. CD133 expression, which is associated with progenitor/stem cells, regeneration, differentiation or metabolism (reviewed in^28^), was found to be increased for BM-MSCs compared to other MSCs sources. CD146 is a key cell adhesion protein in vascular endothelial cell activity and angiogenesis and has recently emerged as an attractive candidate for identifying undifferentiated MSCs^29^. In this study, positivity for CD146 was found approximately in 30% of WJ-MSCs, but below 5% of BM-MSCs and AT-MSCs. The expression of embryonic stem cell marker SSEA-4 has previously been reported for BM-MSCs^30,31^. We observed the expression of SSEA-4 in 60% of BM-MSCs and WJ-MSCs, but only in 10% of AT-MSCs. These results are not consistent with a previous report showing the absence of SSEA-4 expression in umbilical cord-derived MSCs^31^, and might be explained by differences in isolation and expansion methods. In contrast, the expression of MSCA-1 was completely absent in WJ-MSCs cultures, while more than 90% of BM- and AT-MSCs expressed this MSC marker. The absence of MSCA-1 expression in WJ-MSCs could be associated with oxygen levels used during cell expansion^32^. For example, Devito *et al*. showed a significant increase in the number MSCA-1^+^ cells within a WJ-MSCs population after *in vitro* expansion in low oxygen conditions^33^.

The proliferation rate of MSCs differed depending on the tissue source. Compared to adult MSCs, WJ-MSCs showed the highest proliferation activity, which confirms the previously reported data^34–36^. The average population doubling time (PDT) for WJ-MSCs was shorter than 24 hrs and stable for at least 5 passages. Similar observations have been reported by Lu *et al*.^34^. The shorter PDT of umbilical cord MSCs compared to AT-MSCs has been shown by Choudhery *et al*.^37^. A higher proliferation capacity of WJ-MSCs compared to BM- or AT-MSCs has also been demonstrated in other studies^35,36,38^. In the current study, we have also revealed differences in proliferation activity between BM- and AT-MSCs. From passage 2, BM-MSCs showed a longer PDT and lower cPD compared to other cell lines. Similar observations have been reported by Kern *et al*.^39^, showing that BM-MSCs possessed the lowest proliferation capacity compared to AT-MSCs or umbilical cord blood MSCs. Dmitrieva *et al*. showed that although BM-MSCs and AT-MSCs exhibited similar proliferation rates, BM-MSCs entered senescence after passage 4, while most of the AT-MSCs did not show senescence up to passage 8^40^.

The immunomodulatory activity of MSCs, *i.e*. the effect of MSCs on the proliferation of PHA-activated PBMC, was analysed by co-culture in contact or transwell systems. The inhibitory activity of BM-MSCs (MSC:PBMC ratio 1:20 and 1:5 in contact co-culture and transwell, respectively) was significantly higher compared to AT- or WJ-MSCs. There are numerous reports showing both similarities and differences in the immunomodulatory properties of MSCs derived from different human sources. In a recent review by Mattar and Bieback, the authors thoroughly analysed reports on the immunogenicity and immunomodulatory properties of MSCs derived from BM, AT, umbilical cord or placenta^41^. The vast majority of currently published data has shown no significant differences in the immunomodulatory potential of MSCs derived from alternative sources, but results have varied greatly due to the differences in experimental protocols or characteristics of MSC populations^41^. We expect that cell culture conditions (serum-free, FBS-/PL-based or chemically-defined), duration of MSC culture prior to assay, or ratio in MSC to PBMC populations in the co-culture system, may significantly affect the outcomes of the immune studies. In our hands, independently of cell source, MSCs were capable of effectively inhibiting the PHA-stimulated proliferation of PBMC, in both direct contact and non-contact transwell co-culture systems. Although the non-contact co-culture resulted in lower immunosuppression, it confirms the high paracrine activity of MSCs. We have observed a relatively higher expression of *PDL1* gene in BM-MSCs that may be connected with the higher immunomodulatory activity of these cells in co-culture with PHA-stimulated PBMC^42,43^.

The paracrine activity of MSCs is associated with the release of growth factors, or cytokines. We mainly focused on the neuroregenerative and neurotrophic potential of MSCs, in an attempt to reveal differences and similarities among cells of different tissue origin. We analysed the MSC secretome composition and determined a broad spectrum of growth factors relevant to neural regeneration, independently of the tissue of origin. BDNF promotes the survival and differentiation of neuronal tissue by acting on receptor kinases^44^ and is considered one of the main factors involved in neural tissue repair^45–47^. We observed a significant difference in BDNF production between BM-MSCs and WJ-MSCs. Similar differences were observed also for NGF, HGF and FGF-2, which also play a key role in neuroprotection and support neuronal regeneration^48–52^. The lowest production of HGF and FGF-2 was indicated in BM-MSC cultures. On the other hand, we observed a significantly higher production of VEGF-A by BM-MSCs compared to AT- or WJ-MSCs. It has previously been shown that VEGF-A and HGF signalling pathways modulate each other^53,54^. This is in line with our results, where the highest HGF expression observed in WJ-MSCs cultures coincided with the lowest VEGF-A levels.

Although we observed different secretome profiles of MSCs depending on the source of cell isolation, neurotrophic/neuroprotective factors were detected in all secretomes, independently of the MSC origin. Attempting to detect the most important factors which may positively affect neural regeneration or provide protective action towards neural cells after cell transplantation, we applied functional *in vitro* assays using a MSC-CM. Independently of cell source, the MSC-derived CM provided an anti-apoptotic effect in SPC-01 neural stem cells after H_2_O_2_ treatment that mimics oxidative stress. It has previously been shown that BM-, AT-MSCs and umbilical cord perivascular cells produce proteins with potential antioxidative functions, and play a protective role during ROS-associated cell damage^55^.

During the culture of DRG adult neurons in the presence of MSC secretomes we observed a significant improvement of neurite growth. Although no statistically significant differences were detected among the MSC sources, the stimulatory activity of BM-MSC-derived secretome tended to be lower. Recently, Assunção-Silva *et al*.^56^ showed that the conditioned medium obtained from AT-MSCs induced significantly more neurite extensions from DRG explants relative to a BM- or umbilical cord perivascular cell-derived medium. Since in our study the CM was obtained in serum starvation conditions, we hypothesized that such an environment may affect the paracrine capacity of cells. We found that MSCs, independently of the tissue of origin, responded to nutrient limitation by up- or down-regulation of several genes. However, in many cases statistical significance was not achieved due to donor variabilities. We indicated a significant reduction in metabolic activity and *BDNF* gene expression in BM-MSCs in response to serum starvation. Correspondingly, the level of BDNF in BM-MSC secretome was also lowest among the MSC sources. It has been proposed that BDNF is the most important factor inducing axonal outgrowth^45^. In our study we could not detect any significant differences between the trophic effect of BDNF and other growth factors tested (FGF-2 and HGF, see Supplementary information, Fig. S3). However, it should be mentioned that the stimulatory effect of individual growth factors on neurite outgrowth appeared to be less pronounced compared to secretome cocktails produced by MSCs (around 1.65–1.96-fold increase in growth factor groups, compared to 7.2–18.5 times’ increase in MSC-derived secretomes). Thus, we believe the stimulatory effect of an MSC-derived CM towards DRG neurons may be associated with the combined action of the cocktail of growth factors present in MSC secretome. The tendency for the lower activity of BM-MSC-derived CM may be associated with the lower content of the aforementioned growth factors (HGF, BDNF and FGF-2) compared to other cell lines, which may be caused by the higher sensitivity of BM-MSCs to nutrient limitations. It is known that the transplantation of cells is accompanied by a variety of stress factors, including limited nutrient supply, and the response of cells towards such factors during at least the first 24 hrs may partly reflect the initial therapeutic action of cells post injection. The reduced metabolic activity and significant decrease in the expression of *BDNF* in response to serum starvation may result in the lower neurotrophic capacity of these cells and should be considered in selecting the MSC source or cell culture conditions in further studies.

Therefore, in this study, we performed a thorough analysis of the properties of *in vitro* expanded MSCs, depending on their origin (Table 1). We confirmed the non-equality of MSCs derived from different sources, regarding their growth kinetics, immunophenotype, immunomodulatory properties, gene expression and paracrine activity. We showed that BM-MSCs cultured in proposed clinically-relevant conditions are characterized by the lowest proliferation capacity and the highest sensitivity to the stress microenvironment, which should be considered in further applications. On the other hand, BM-MSCs displayed the highest immunomodulatory activity compared to AT- and WJ-MSCs. Despite the differences in growth factor secretion depending on the source of cell isolation, the MSC-derived secretome has a pronounced neurotrophic potential to stimulate the *in vitro* sprouting of adult DRG dissociated neurons. Special attention should be paid to the response of MSC cultures to the conditions that occurred *in vivo* after transplantation, including oxygen and nutrient restrictions (shown in this study). The response of MSCs to pro-inflammatory environment, which occurs in the damaged tissues is another very important point of consideration. In our primary evaluation of the response of MSCs from different sources towards the pro-inflammatory factors (TNF-α and IFN-γ) *in vitro* we detected remarkable increase in the secretion of IL-6, IDO, sICAM-1, sVCAM-1 and BDNF (Supplementary information, Fig. S4), confirming other reported data^57,58^. The further thorough investigation of the response of MSCs towards different cell pre-adaptation and pre-treatment strategies is needed to expand the spectrum of therapeutic properties of cells and optimize the manufacturing processes. Overall, our study provides important information for the transfer of basic MSC research to clinical-grade manufacturing and therapeutic applications.

## Methods

Unless otherwise indicated, chemicals were purchased from Sigma-Aldrich (St Louis, MO, USA). All donors of human tissue provided their written informed consent prior to any intervention. Human tissues and cells were donated anonymously. All experiments on the animals were performed in accordance with the European Communities Council Directive of 24th November 1986 (86/609/EEC), regarding the use of animals in research. All studies involving human and animal tissues or cells were approved by the Ethical Committee of the Institute of Experimental Medicine Academy of Sciences Czech Republic, Prague. All methods were performed in accordance with the relevant guidelines and regulations.

### Isolation and culture of cells

#### Bone marrow

Human bone marrow (BM) was obtained from the iliac crest of healthy donors (N = 7) that were either subjects in a clinical trial or undergoing orthopaedic surgery for other than BM related problems. The unused surplus of mononuclear cell fraction obtained from BM after erythrocyte depletion using Gelofusine (B. Braun, Melsungen AG, Germany) sedimentation were supplied by Bioinova, Ltd. (Prague, Czech Republic) and processed as described previously^59,60^. Mononuclear cells were cultured in a complete culture medium (CCM) containing α-MEM (LONZA, Basel, Switzerland), 5% pooled PL (Bioinova, Ltd.), and 10 µg/ml gentamicin (Sandoz, Holzkirchen, Germany) at 37 °C, in a humidified atmosphere, containing 5% CO_2_ with regular media changes twice a week. After reaching near-confluence, cells were harvested using the 0.05% Trypsin/EDTA solution (Life Technologies, Carlsbad, CA, USA) and reseeded onto a fresh plastic surface (Nunc, Roskilde, Denmark) at a density of 5 × 10^3^ cells/cm^2^. Cells derived at passage 3 were used for the evaluation of immunophenotype, differentiation properties, gene expression and secretome profile in normoxic and hypoxic (5% O_2_) conditions.

#### Adipose tissue

Adipose tissue (AT) samples were obtained from healthy volunteers (N = 9) who had undergone liposuction procedures for aesthetic reasons and processed as previously described^61^. Briefly, the lipoaspirate was repeatedly washed in phosphate-buffered saline (PBS; IKEM, Prague, Czech Republic) and enzymatically digested by collagenase (0.3 PzU/ml, Collagenase NB 6 GMP; Serva Electrophoresis GmbH, Heidelberg, Germany) at 37 °C for 2 hours with constant shaking. After centrifugation at 200 × g for 10 min, the stromal vascular fraction was washed twice with PBS and cultured under the same conditions as previously described for BM.

#### Umbilical cord tissue

Discarded human umbilical cords (UC) were obtained from healthy full-term neonates (N = 15) after spontaneous delivery. About 10 cm of UC was aseptically stored in sterile PBS (IKEM) with antibiotic–antimycotic solution (AA) at 4 °C and transported to the laboratory within 24 hours. After washing several times in PBS-AA and briefly washing in 10% Betadine (EGIS Pharmaceuticals PLC, Budapest, Hungary), blood vessels were removed and the remaining Wharton’s jelly tissue (WJ) was chopped into small fragments (1–2 mm^3^) and weighed. WJ-MSCs were isolated from fragments (1 g) by digestion in PBS-AA solution containing 0.26 U/ml Liberase^TM^ (Roche Custom Biotech, Mannheim, Germany)^62^ and 1 mg/ml hyaluronidase at 37 °C with constant shaking for 2 hours. After the removal of undigested fragments with 40-µm cell strainers, cells were subjected to centrifugation (450 × g, 10 minutes), diluted in a complete culture medium and cultured as described above for BM.

#### Proliferation and cell yield

The proliferation capacity of MSCs obtained from different tissues (N = 7 for BM-MSCs, N = 9 for AT-MSCs and N = 15 for WJ-MSCs) was evaluated from passage one (P1) up to passage ten (P10). Cells (5 × 10^3^ cells/cm^2^) were plated in duplicate into a 6-well culture plate (TPP Techno Plastic Products AG, Trasadingen, Switzerland) and harvested at 90% of confluence 3–7 days later. The number of harvested viable cells was counted by Trypan blue dye exclusion methods using a Bürker chamber, and 5 × 10^3^ cells/cm^2^ were reseeded into new plates.

As proposed by Brudet *et al*.^63^ and Greenwood *et al*.^64^ cell proliferation was assessed in population doublings (PD). At each passage the PD and population doubling time (PDT) were calculated according to these formulas:

$$PD=[\log (Nh)-\,\log (Ns)]/\log \,2,$$

$$PDT=CT\ast log2/[log(Nh)-log(Ns)],$$

where Ns and Nh are the number of seeded and harvested cells, respectively and CT is the cell culture time (hours). The cumulative population doubling (cPD) level was calculated by summation of the PD values obtained for each passage.

### Immunophenotype

To characterize MSCs derived from different sources (N-7 for each tissue of MSC isolation), the following conjugated antibodies were used: CD10 FITC, CD14 FITC, CD29 APC, CD44 FITC, CD45 PE-Cy7, CD73 PE, CD90 APC, CD105 AF488, CD146 PE, CD271 (NGFR) PE, (Exbio Antibodies, Vestec u Prahy, Czech Republic), VEGFR2, SSEA-4 PE (BioLegend, San Diego, CA, USA), CD133 PE, MSCA-1 APC (Miltenyi Biotec Inc., Bergisch Gladbach, Germany), CD34 PE-Cy5, HLA-ABC, HLA-DR (BD Bioscience, San Diego, CA, USA) and CD235a PE (Abcam, Cambridge, UK). As negative controls, mouse IgG1 and IgG2a conjugated with FITC, PE and PE-Cy5 (Dako Cytomation, Glostrup, Denmark, BD Bioscience, San Diego, CA, USA) were used. Briefly, cells from passage 3 were sampled in designated tubes of at least 250 000 cells in 100 µl, and incubated with the appropriate amount of antibodies according to the manufacturer’s recommendation. Cells were then washed with PBS, fixed in 4% paraformaldehyde overnight and at least 10 000 cells were recorded on BD FACSAria^TM^ Cell Sorter, BD LSR II (BD Bioscience, San Diego, CA, USA) or Apogee A-50 Micro flow cytometer (Apogee Flow Systems, Hertfordshire, UK). The obtained data were then analysed using FlowLogic™ Software (Inivai™ Technologies, Mentone, Australia).

### Immunomodulatory properties

The immunomodulatory properties of MSCs derived from different sources (N = 3 in duplicates for each tissue of MSC isolation) were assessed by the ability of MSCs to suppress the phytohemagglutinin (PHA)-induced proliferation of human peripheral blood mononuclear cells (PBMC) in a contact or non-contact co-culture^60^. PBMC from at least 3 different human peripheral blood donors (obtained from the Institute of Hematology and Blood Transfusion, Prague, Czech Republic) were isolated by Ficoll-Paque (Miltenyi Biotec Inc., ρ = 1.077 g/ml) gradient centrifugation, according to the manufacturer’s instructions. For contact co-culture experiments, MSCs were seeded into separate wells of 96-well plates in the densities 5 × 10^4^; 2 × 10^4^; 10^4^; 5 × 10^3^ and 10^3^ cells per well to achieve the PBMC:MSC ratios 2:1; 5:1; 10:1; 20:1 and 40:1, respectively. After 2 hrs of culture and cell attachment, MSCs were treated with mitomycin C (10 µg/ml) for 2 hrs to arrest cell proliferation. After 3 washes with PBS, MSCs were cultured for 24 hrs before the co-culture experiment. The culture medium was discarded and 10^5^ PBMC were seeded into each well (200 µl/well) in RPMI-1640 medium, supplemented with 10% FBS (BIOSERA, Nuaille, France) and PHA (500×, Thermo Fisher Scientific Inc., Waltham, MA, USA). Non-contact co-cultures of MSCs and PBMC were prepared using transwell culture inserts (Merck, Darmstadt, Germany). Briefly, MSCs were seeded into separate wells of a 24-well plate in concentrations of 10^5^, 5 × 10^4^, 2 × 10^4^ and 10^4^ cells per well to achieve the final PBMC:MSC ratios 1:1; 1:2; 1:5 and 1:10. Cells were treated with mitomycin C, as previously described. After 24 hrs transwell inserts with 10^5^ PBMC/per insert in 200 µl of FBS-supplemented RPMI-1640 culture medium were placed in the MSC-containing wells. In each experiment MSCs from at least 3 donors were co-cultured with PBMC from 3 donors. After 96 hrs of co-culture, the proliferation of PBMC was assessed by Alamar Blue (AB, Thermo Fisher Scientific inc.) assay. Briefly, cells in each well or transwell insert were thoroughly resuspended and 100 µl of PBMC were transferred into a fresh well of a 96-well plate. Then, 200 µl of 10% AB solution was added to each well and cells were incubated for 4 hrs at 37 °C, 5% CO_2_, and 95% humidity. The fluorescence level of reduced AB was assessed using TECAN GENios microplate reader (Tecan, Männedorf, Switzerland) with an excitation wavelength of 550 nm and an emission wavelength of 590 nm. The ratio between the fluorescence of experimental and blank sample (without cells) was used as the AB value. The index of PBMC proliferation was determined as the ratio in AB values of PHA-stimulated (alone or in co-culture) and non-stimulated PBMCs.

### Gene expression

Changes in the mRNA expression were determined by quantitative real-time PCR (qPCR). The following human genes were selected to perform the multi-sided screening of different cell functions: chemoattractants (*CXCL12*, *CXCR4*), transcription factors (*TWIST*), genes associated with immunomodulation and inflammation (*IDO1, COX2, PDL1, TGFB1, IL6, IL10, IL12A*), adhesion molecules (*ICAM, VCAM, NCAD*), as well as genes related to angiogenesis and neuroregeneration (*HGF, BDNF, TUBB3, VEGFA*). RNA was isolated using the RNeasy Mini Kit (Qiagen, Hilden, Germany) and RNA amounts were quantified using NanoPhotometer® P330 (Implen, Munich, Germany). The isolated RNA was reverse transcribed into complementary DNA using the Transcriptor Universal cDNA Master (Roche) and T100TM Thermal Cycler (Bio-Rad, Hercules, USA). The qPCR reactions were performed using cDNA solution, FastStart Universal Probe Master (Roche) and TaqMan® Gene Expression Assays (Thermo Fisher Scientific Inc.) (Supplementary information, table S1). The qPCR was carried out at a final volume of 10 µl containing cDNA equivalent of 65 ng of extracted RNA. Amplification was performed on the real-time PCR cycler (StepOnePlus^TM^, Life Technologies). All amplifications were run under the same cycling conditions: 2 min at 50 °C, 10 min at 95 °C, followed by 40 cycles of 15 s at 95 °C and 1 min at 60 °C. All samples were run in duplicate and a negative control (water) was included in each array, with GAPDH as a reference gene. The results were expressed as 2^(−∆Ct) changes of ∆Ct values.

### Metabolic activity of MSCs under serum starvation (PL-free) conditions

MSCs from different sources (N = 3 in duplicates for each of MSC isolation source) were expanded for 5 days in CCM and treated for 2 hrs with mitomycin C (10 µg/ml) at 37 °C to arrest cell proliferation. After extensive washing with CCM, the medium was changed either to CCM or PL-free medium without any supplements. MSCs were cultured for 24 hrs, followed by the addition of Alamar Blue (AB) to reach a10% concentration. After 4 hrs of additional incubation, the fluorescence level of the reduced AB was assessed using a TECAN GENios microplate reader (Tecan) with an excitation wavelength of 550 nm and an emission wavelength of 590 nm^65^. The ratio between the fluorescence of experimental and blank sample (same medium without cells) was used as the AB value. The data was presented as relative fluorescence units (RFU).

### Analysis of MSC secretome

The conditioned medium for secretome analysis was collected after 24 hrs cultivation of WJ-MSCs, BM-MSCs and AT-MSCs in a PL-free medium to detect factors released specifically by MSCs rather than present in PL supplement (N = 3 in duplicates for each tissue of MSC isolation). Briefly, MSCs at passage 3 were seeded at the density 4000 cells/cm^2^ and cultured in CCM to an 80% confluence. Then, the CCM was removed and after 3 washes with PBS replaced by PL-free α-MEM. After 24 hours, the conditioned medium was collected and centrifuged at 1,500 rpm for 10 min, filtered through a 0.22 µm filter and immediately stored at −80 °C. Concentrations of cytokines and growth factors including BDNF, SDF-1α, VEGF-A, sICAM-1, sVCAM-1, FGF-2, HGF, MCP-1, IL-6, NGF were assessed by Luminex®-based multiplex ProcartaPlex® Immunoassay (Thermo Fisher Scientific Inc.), as previously described^66^, using ten human Simplex™ Kits for individual analyses and one Basic Kit according to the manufacturer’s instructions. All samples were analysed in duplicate. All experiments were repeated 3 times with different donors. The data were obtained using a Luminex® 200™ Instrument (EMD Millipore, Billerica, MA, USA) operated by xPonent software 3.1.871.0 (Luminex Corporation, Austin, TX, USA) and the data were processed by drLumi package^67^ in R statistical environment^68^. The median fluorescence intensities (MFI) of cytokine and growth factor standard dilutions were used for standard curve fitting using 5-parameter logistic regression (SSL5) with 4-parameter logistic regression (SSL4) as a fallback in case the SSL5 model did not converge. Cytokine concentrations in samples were derived from measured MFIs using fitted standard curves.

### Dorsal root ganglion neural culture and neurite outgrowth in the presence of an MSC conditioned medium

Dorsal root ganglia (DRGs) were dissected from adult male Wistar rats (3 months). The neurons were dissociated with 0.2% collagenase and 0.1% trypsin and then centrifuged through 15% bovine serum albumin (BSA) in DMEM (Thermo Fisher Scientific Inc.). DRGs were cultured in 24-well plates on coverslips coated with poly-D-lysine (20 µg/ml) and laminin (1 µg/ml) in DMEM, supplemented with penicillin–streptomycin–fungizone (1%, Lonza) and mitomycin C (0.25 µg/ml) mixed in 1:1 ratio with CM (prepared in the absence of PL) or α-MEM (N = 3 in duplicates for each of the MSC source). After 24 hrs of culture, the cells were fixed with 4% paraformaldehyde in PBS and stained with anti-beta III tubulin (Abcam) and goat anti-mouse IgG Alexa Fluor® 488 (Life Technologies). Images were acquired on Zeiss Axioskop 2 Plus fluorescent microscope. An analysis of the percentage of beta III tubulin positive area was calculated using ImageJ software 1.8.0_112 and related to the positive area of DRGs cultured in the control medium (α-MEM without supplements).

### SPC-01 neural stem cell culture and induction of oxidative stress

A human neural conditionally immortalized cell line (SPC-01) was prepared according to the protocol described previously^69^. A retroviral vector encoding the immortalizing construct (c-mycERTAM) was used to immortalize the cells from 10-week-old human fetal spinal cords. Cells were cultured in laminin-coated 96-well plates in growth media composed of DMEM /F12 supplemented with human serum albumin (HSA; 0.03%; Baxter Healthcare Ltd., Norfolk, UK), human transferrin (100 μg/ml), L-glutamine (2 mM), putrescine dihydrochloride (16.2 μg/ml), sodium selenite (selenium; 40 ng/ml), human insulin (5 μg/ml), epidermal growth factor (EGF; 20 ng/ml, PeproTech, London, UK), progesterone (60 ng/ml), and 4-OHT (100 nM), and fibroblast growth factor 2 (FGF-2; 10 ng/ml; PeproTech). When the cells reached confluence, the medium was changed to serum-free α-MEM. The concentrated solution of H_2_O_2_ was added to the cell culture wells to achieve a final concentration of 250 µM. After 2 hrs incubation at 37 °C, the medium was changed to either serum-free α-MEM (negative control) or MSC-derived CM, prepared in the absence of PL. Cells were cultured for 24 hrs and their viability was determined by AB test, as described previously. The recovery of SPC-01 cells was calculated as a ratio of H_2_O_2_ treated to non-treated cells.

### Statistical analysis

The data are presented as mean ± standard error of mean (SEM). In different parts of the study statistical significance was analysed using the following methods: For cell proliferation, flow cytometry, cytokine production and DRG-neurite outgrowth assay – One-way ANOVA with a Student–Newman–Keuls (SNK) post hoc pair-to-pair test. For gene expression studies - the differences between dCt levels were analysed by one-way ANOVA; all pair-wise multiple comparisons were analysed using the SNK method. For immunomodulatory properties, DRG-neurite outgrowth assay and analysis of the effect of MSC-derived CM on the recovery of neural stem cells after H_2_O_2_ treatment - a Mann-Whitney nonparametric test; for metabolic activity analysis a paired Student’s t-test; Data were analysed using SigmaPlot V13 (Systat Software Inc., San Jose, CA, USA); Differences were considered statistically significant if p < 0.05.

## Supplementary information

Supplementary information.

## Data Availability

The datasets generated and/or analysed during the current study are available from the corresponding author on reasonable request.
