# Supplementary material for: A Comparative Analysis of Multipotent Mesenchymal Stromal Cells derived from Different Sources, with a Focus on Neuroregenerative Potential
Source: Sci Rep. 2020 Mar 9;10:4290. doi: 10.1038/s41598-020-61167-z (PMC7062771; doi:10.1038/s41598-020-61167-z)
Supplement: Supplementary file 1 — Supplementary information. [file 41598_2020_61167_MOESM1_ESM.pdf]

Supplementary information

COMPARATIVE ANALYSIS OF MULTIPOTENT MESENCHYMAL STROMAL CELLS DERIVED FROM DIFFERENT SOURCES WITH A FOCUS ON NEUROREGENERATIVE POTENTIAL

Yuriy Petrenko, Irena Vackova, Kristyna Kekulova, Milada Chudickova, Zuzana Koci, Karolina Turnovcova, Helena Kupcova Skalnikova, Petr Vodicka, Sarka Kubinova

1. Differentiation properties of MSCs derived from different sources

Materials and methods

To promote osteogenic and adipogenic differentiation, minimally three different BM-MSCs, AT-MSCs and WJ-MSCs samples at passage three (P3) were initially seeded in six-well plates (60,000 cells/well) in a complete culture medium (CCM) at 37°C and 5% CO_2 in a humidified atmosphere containing 95% air. After reaching 90% confluence, the culture medium was removed and the appropriate differentiation media were added to the cultures. To induce osteogenic differentiation, the cells were cultured for minimally 3 weeks in an induction medium composed of α-MEM, supplemented with with 10% fetal bovine serum (Biosera), 50 U/ml penicillin, 50 µg/ml streptomycin, 10 mM glycerol-2-phosphate, 0.1 µM dexamethasone and 100 µM L-ascorbic acid. The medium was changed twice weekly. Osteogenesis was assessed by detection of calcified extracellular matrix deposits using Alizarin Red S staining.

For adipogenic differentiation, near-confluent cells were grown in a medium composed of α-MEM, containing 10% FBS, 0.5 mM 3-isobutyl-1-methylxanthine, 0.1 µM dexamethasone, 0.1 mM indomethacin, 50 U/ml penicillin, 50 µg/ml streptomycin and 10 µg/ml insulin. Adipogenesis was assessed using conventional Oil Red O staining to visualize lipid droplets in the cytoplasm.

To induce chondrogenic differentiation, 6-7 × 10^5 cells were pelleted for 5 minutes at 250 x g in polypropylene conical 15-ml Falcon tubes and cultured in 1 ml of serum-free medium composed of α-MEM, supplemented with 10 ng/ml human transforming growth factor β1 (TGF-β1), 1% ITS (insulin, transferrin, sodium selenite), 0.1 µM dexamethasone, 50 µM L-ascorcbic acid, 50 U/ml penicillin, and 50 µg/ml streptomycin. The medium was replaced twice weekly for 3-4 weeks. Pellets were fixed in 4% paraformaldehyde for 10 minutes, followed by routine embedding, sectioning, and staining with Alcian blue and Nuclear Fast Red to detect sulphated glycosaminoglycan deposition and pellet morphology. All samples were evaluated by light microscopy (Zeiss Axioskop 2 plus microscope, Oberkochen, Germany).

Results

The induction of adipogenic, osteogenic or chondrogenic differentiation resulted in corresponding changes in cell morphology and the appearance of specific signs of differentiation: the accumulation of lipid droplets in adipogenic cultures, matrix mineralization in osteogenic and accumulation of extracellular matrix in chondrogenic conditions (Fig. S1).

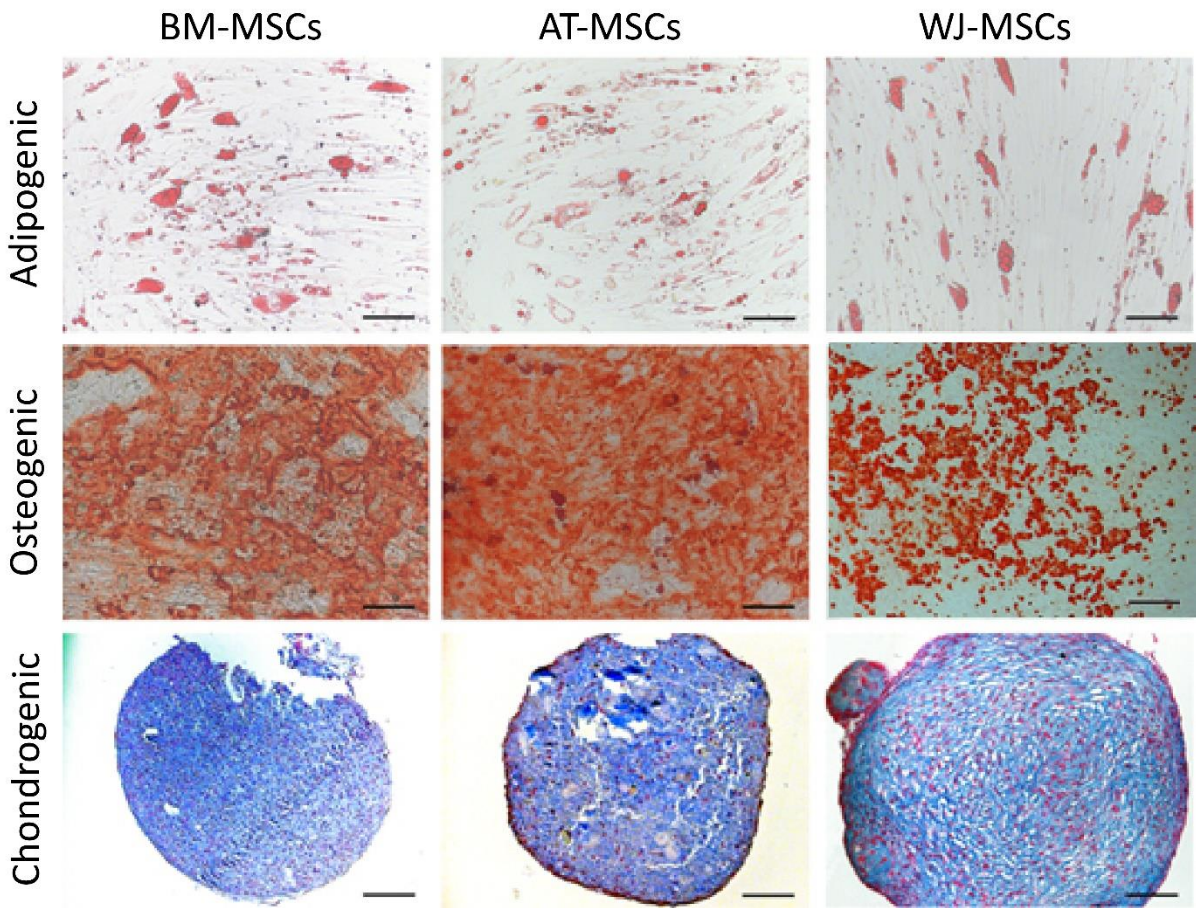

Fig. S1. The multi-lineage differentiation of MSCs derived from different sources (scale bar 200 µm).

2. Migratory capacity

Materials and methods

To perform a migration assay, xCELLigence RTCA DT Instrument (Acea Biosciences Inc. San Diego, CA, USA) was used. Prior to the migration assay, cells were left for 24 hours in a media

without PL. On the day of the experiment, a medium with SDF-1α (20 ng/ml) was placed into a lower chamber of CIM-Plate 16, and MSCs were then pipetted into the upper chamber (10^5 cells in 100 µl of media without supplements). The recorded impedance signal at 6 hours was normalized to migration towards supplement-free media and was then used to assess the rate of cell migration. The assay was repeated five times.

Results

Migratory capacity is one of the essential MSC properties that enables their trafficking and thus allows them to act at the site of injury. In a transwell culture, we confirmed the capacity of all MSCs to migrate towards SDF-1α. The migration rate did not differ between individual cell types (Fig. S2).

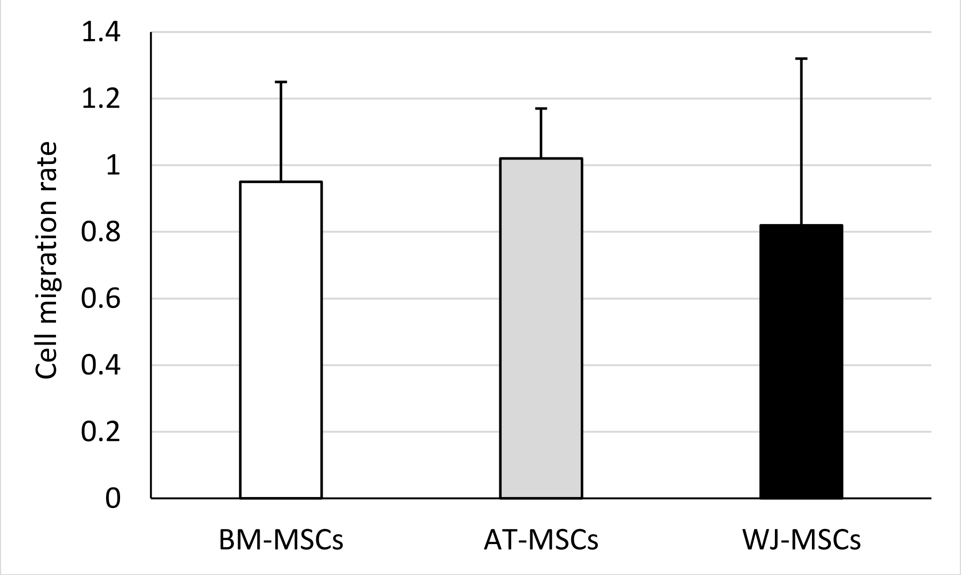

Fig. S2. Migratory capacity towards SDF-1α of MSCs derived from different sources (mean ± SEM, N=5).

3. Gene expression

Table S1

TaqMan® Gene Expression Assays (Thermo Fisher Scientific).

CXCL12	chemokine (C-X-C motif) ligand 12	Hs03676656_mH
CXCR4	C-X-C chemokine receptor type 4	Hs00607978_s1
IDO	Indoleamine 2,3-dioxygenase	Hs00984148_m1
COX2	Cyclooxygenase-2	Hs00153133_m1

PDL1	Programmed death-ligand 1	Hs00204257_m1
HGF	Hepatocyte growth factor	Hs00300159_m1
TGFB1	Transforming growth factor beta 1	Hs00998133_m1
TWIST	Twist Family BHLH Transcription Factor 1	Hs01675818_s1
IL6	Interleukin 6	Hs00174131_m1
IL10	Interleukin 10	Hs00961622_m1
IL12A	Interleukin-12 subunit alpha	Hs01073447_m1
ICAM	Inter-cellular adhesion molecule	Hs00164932_m1
VCAM	Vascular cell adhesion molecule	Hs01003372_m1
NCAD	N-Cadherin	Hs00983056_m1
VEGFA	Vascular endothelial growth factor A	Hs00900055_m1
BDNF	Brain-derived neurotrophic factor	Hs02718934_m1
TUBB3	Tubulin Beta 3 Class III	Hs00801390_s1

4. The effect of neurotrophic growth factors on the neurite outgrowth area of rat DRG-neurons

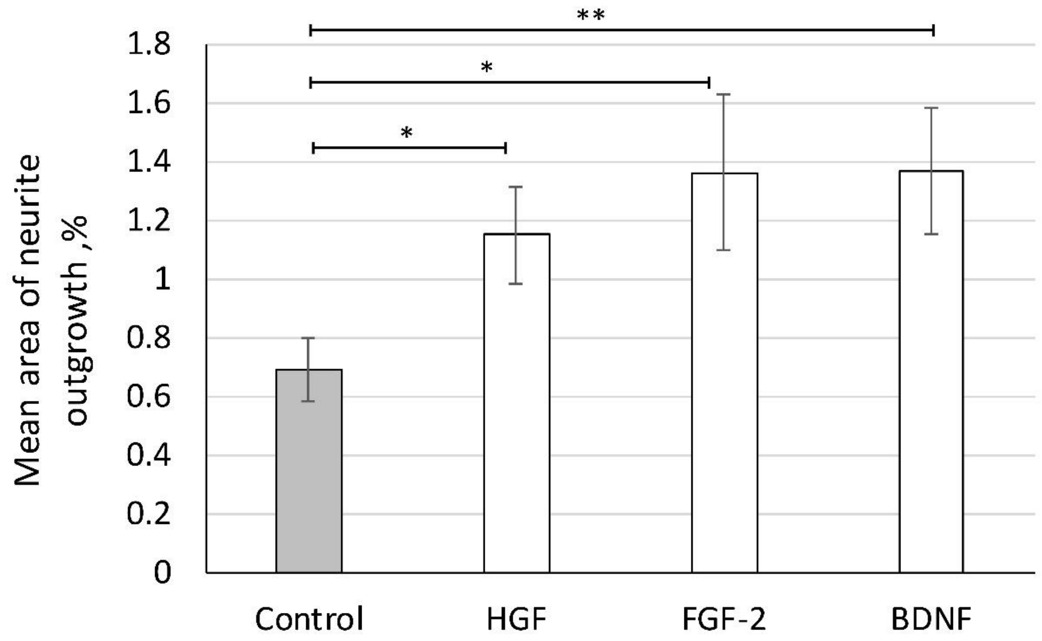

Fig. S3. The effect of neurotrophic growth factors (10 ng/ml) on the neurite outgrowth area of rat DRG-neurons (mean ± SEM).
*, ** - values are significantly different (*-p<0.05, ** - p<0.01) between the indicated groups. One-way ANOVA with Dunn's post hoc pair-to-pair test (N=3 in duplicates).

5. The effect of cytokine priming *in vitro* on secretome composition of MSCs derived from different sources

The response of MSCs to pro-inflammatory environment, which occurs in the damaged tissues is very important point for translational MSC research. It is known that under the pro-inflammatory conditions the MSCs become activated with corresponding upregulation of immunomodulatory genes, changes in paracrine activity, migration and survival [1-3]. Here we show the primary evaluation of the composition of MSC secretome obtained after cell priming with the pro-inflammatory factors (TNF-α and IFN-γ) *in vitro*.

Materials and methods

The conditioned medium for secretome analysis was collected after 24 hrs cultivation of WJ-MSCs, BM-MSCs and AT-MSCs in a PL-free medium to detect factors released specifically by MSCs rather than present in PL supplement. However, to avoid the serum starvation associated changes in gene expression and secretory activity, shown previously, the culture medium was additionally supplemented with 1% Insulin-transferrin-selenium supplement (ITS, Thermo Fisher Scientific Inc.). To reduce the donor-associated variabilities, MSC suspensions obtained from 3 different donors were pooled into one vial and seeded at the density 5000 cells/cm^2. The same procedure was prepared for each of the tissue of cell isolation. MSCs were cultured in CCM to an 80% confluence. Then, the CCM was removed and after 3 washes with PBS replaced by PL-free α-MEM, containing ITS supplement and TNF-α (10 ng / ml) and IFN-γ (10 ng / ml)[4]. After 24 hours, the conditioned medium was collected and centrifuged at 1,500 rpm for 10 min, filtered through a 0.22 µm filter and immediately stored at -80°C. Concentrations of cytokines and growth factors including BDNF, SDF-1α, IDO, VEGF-A, sICAM-1, sVCAM-1 and IL-6 were assessed by Luminex®-based multiplex ProcartaPlex® Immunoassay (Thermo Fisher Scientific Inc.), using seven human Simplex™ Kits for individual analyses and one Basic Kit according to the manufacturer's instructions. All samples were analysed in duplicate. The data were obtained using aLuminex® 200™ Instrument (EMD Millipore, Billerica, MA, USA) operated by xPonent software 3.1.871.0 (Luminex Corporation, Austin, TX, USA) and the data were processed by drLumi package in R statistical environment. The median fluorescence intensities (MFI) of cytokine and growth

factor standard dilutions were used for standard curve fitting using 5-parameter logistic regression (SSL5) with 4-parameter logistic regression (SSL4) as a fall back in case the SSL5 model did not converge. Cytokine concentrations in samples were derived from measured MFIs using fitted standard curves.

Results

The remarkable increase in the secretion of IL6, IDO, sICAM-1, sVCAM-1 and BDNF was detected after cytokine treatment, independently on the source of cell isolation (Fig. S4). These results confirm the previously published data[1,3]. In turn, among the growth factors tested SDF-1α and VEGF-A were secreted constitutively regardless of culture conditions.

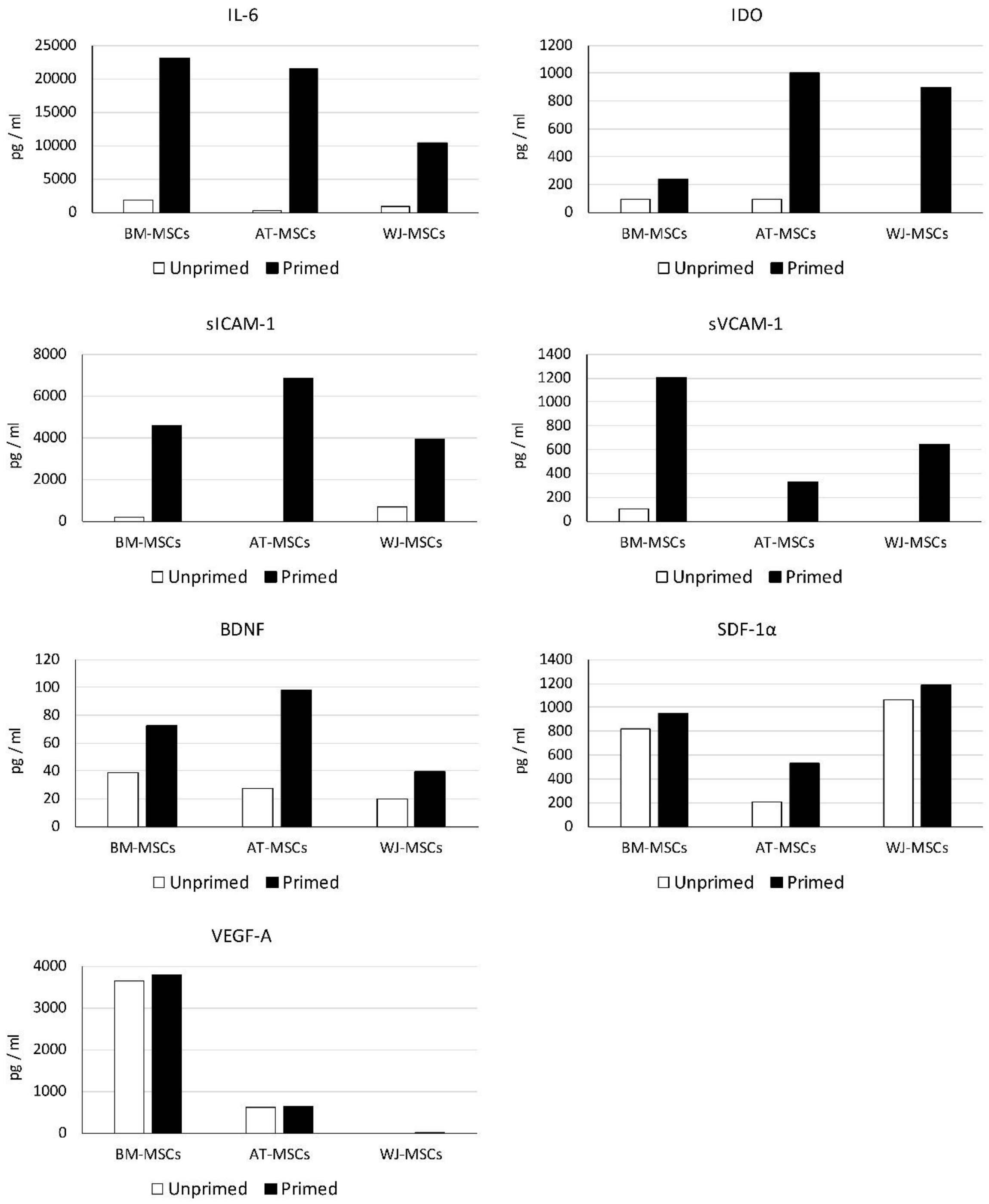

Fig. S4. The effect of TNF-α/IFN-γ treatment on the content of growth factors and cytokines in the conditioned medium produced by MSCs from different tissues.

References

1 Szabo, E. *et al.* Licensing by Inflammatory Cytokines Abolishes Heterogeneity of Immunosuppressive Function of Mesenchymal Stem Cell Population. *Stem Cells Dev* **24**, 2171-2180, doi:10.1089/scd.2014.0581 (2015).

2 Noronha, N. C. *et al.* Priming approaches to improve the efficacy of mesenchymal stromal cell-based therapies. *Stem Cell Res Ther* **10**, 131, doi:10.1186/s13287-019-1224-y (2019).
3 Francois, M., Romieu-Mourez, R., Li, M. & Galipeau, J. Human MSC suppression correlates with cytokine induction of indoleamine 2,3-dioxygenase and bystander M2 macrophage differentiation. *Molecular therapy : the journal of the American Society of Gene Therapy* **20**, 187-195, doi:10.1038/mt.2011.189 (2012).
4 English, K., Barry, F. P., Field-Corbett, C. P. & Mahon, B. P. IFN-gamma and TNF-alpha differentially regulate immunomodulation by murine mesenchymal stem cells. *Immunology letters* **110**, 91-100, doi:10.1016/j.imlet.2007.04.001 (2007).
